# Factors influencing the length of hospital stay during the intensive phase of multidrug-resistant tuberculosis treatment at Amhara regional state hospitals, Ethiopia: a retrospective follow up study

**DOI:** 10.1186/s12889-020-09324-x

**Published:** 2020-08-08

**Authors:** Koku Sisay Tamirat, Gashaw Andargie, Yaregal Animut Babel

**Affiliations:** 1 Department of Epidemiology and Biostatistics, Institute of Public Health, College of Medicine and Health Sciences, University of Gondar, POB: 196, Gondar, Ethiopia; 2 Department of Health Service Management and Health Economics, Institute of Public Health, College of Medicine and Health Sciences, University of Gondar, POB: 196, Gondar, Ethiopia

**Keywords:** Multidrug-resistant tuberculosis, Length of hospital stay, Ethiopia

## Abstract

**Background:**

The length of hospital stay is the duration of hospitalization, which reflects disease severity and resource utilization indirectly. Generally, tuberculosis is considered an ambulatory disease that could be treated at DOTs clinics; however, admission remains an essential component for patients’ clinical stabilization. Hence, this study aimed to identify factors influencing hospital stay length during the intensive phase of multidrug-resistant tuberculosis treatment.

**Methods:**

A retrospective follow-up study was conducted at three hospitals, namely the University of Gondar comprehensive specialized, Borumeda, and Debremarkos referral hospitals from September 2010 to December 2016 (*n* = 432). Data extracted from hospital admission/discharge logbooks and individual patient medical charts. A binary logistic regression analysis was used to identify factors associated with more extended hospital stays during the intensive phase of multidrug-resistant tuberculosis treatment.

**Result:**

Most patients (93.5%) had a pulmonary form of multidrug-resistant tuberculosis and 26.2% had /TB/HIV co-infections. The median length of hospital stays was 62 (interquartile range from 36 to 100) days. The pulmonary form of tuberculosis (Adjusted odds ratio [AOR], 3.47, 95% confidence interval [CI]; 1.31 to 9.16), bedridden functional status (AOR = 2.88, 95%CI; 1.29 to 6.43), and adverse drug effects (AOR = 2.11, 95%CI; 1.35 to 3.30) were factors associated with extended hospital stays.

**Conclusion:**

This study revealed that the length of hospital-stay differed significantly between the hospitals. The pulmonary form of tuberculosis decreased functional status at admission and reported adverse drug reactions were determinants of more extended hospital stays. These underscore the importance of early case detection and prompt treatment of adverse drug effects.

## Background

Tuberculosis is the leading cause of mortality and morbidity and an increased concern for global health [1–3]. The emergence of drug-resistant strains of *Mycobacterium tuberculosis* further complicated tuberculosis treatment and control efforts worldwide [4]. According to the 2019 World Health Organization (WHO) report, there were about 484, 00 incident cases of MDR/RR-TB and 214, 000 deaths from MDR/RR-TB [5]. Thirty high burden countries carry more than 85% of the world’s drug-resistant tuberculosis (DR-TB) cases [6]. Ethiopia ranked third amongst the high burden countries in Africa, with an estimated 2100 MDR-TB cases annually [7]. Besides, the incidence of susceptible TB was an estimated 165 cases per 100,000 population and ranked 3rd in Africa and 11th in the world, according to the 2019 global TB report [5].

Reports showed that the appropriate treatment of tuberculosis averted about 50 million deaths between 2000 and 2018 [5]. Treatment of drug-resistant tuberculosis is longer and usually lasts 18 to 24 months, while successful treatment outcomes remained around 50% [8]. The standard drug-resistant treatment regimen is a treatment protocol designed based on data from population-based drug-resistance surveys about tuberculosis drugs. Due to the lack of susceptibility tests for all ant-TB drugs, an individualized regimen is less practiced, and the standard regimen is preferable. Thus, all patients in the same group received the same drug regimen based on national guidance and widely approached in Ethiopia [9, 10].

The intensive phase of drug-resistant TB treatment, the first 8–12 months, is when patients suffer from critical disease conditions and drug side-effects [11, 12]. In the initial phase, all the efforts are directed to ensure that patients are clinically stable and adherent to SLDs. Hence, the clinical team’s role at hospitals is more intensive to provide the necessary clinical, adherence, and social support arrangements to enable the patient to be fit enough to be followed at the DOTs clinic.

Moreover, life-threatening adverse drug effects of second-line anti-TB medications led to more frequent and prolonged hospitalizations during this phase of the treatment [13, 14]. Length of hospital stay (LOS) refers to the duration of hospitalization [15, 16]. It reflects several aspects of hospital care, including the complexity of the case, hospital care efficiency, and the nature of hospital policies on admission and discharge [15]. The length of hospital stays can also be an indirect estimator of resource utilization and efficiency within a hospital setting and has direct implications for overall healthcare planning and policy [4, 9, 17]. Prolonged hospital stay in the treatment of DR-TB affects the hospital’s efficiency by increasing resource utilization like increased bed occupancies, frequent physician visits, compromise the quality of care, and creating long waiting lists among patients for admission and treatment [4, 16, 18–20]. A study in South Africa revealed that the mean cost per MDR-TB patient was 17,164 USD, of which 95% were hospitalization costs (buildings, staff, etc.) [17]. Also, in-hospital patient management associated with an increased risk of nosocomial infection transmission that affects the quality of care and treatment outcome [21].

Globally, the reported median length of hospital stay was 90 days in high burden MDR-TB countries during the treatment [22]. Africa is characterized by a high burden of drug-resistant tuberculosis and inefficient and inadequate healthcare facilities for the treatment of the disease. Findings from South Africa showed a LOS median of 144 days [23] and those of Nigeria 135 days [8]. Patient functional status, co-morbidities, extensive lung damage, and adverse drug effects were determinants of prolonged hospital stay in the course of MDR-TB treatment [2, 11, 23–26].

Concerning health facility availability and efficiency, the WHO recommends a conditional ambulatory care model in the standard treatment of drug-resistant tuberculosis [27, 28]. Although Ethiopia is one of the high MDR-TB burden countries, there have been only limited health facilities providing MDR-TB treatments with scarce evidence on length of hospital stay and associated factors.

Therefore, this study aimed to determine the length of hospital stay and identify factors associated with extended hospital stays during MDR-TB treatment. The study could be of paramount importance to clinicians and hospital administrators for efficient planning of drug-resistant tuberculosis treatment programs and resource allocations.

## Methods

### Study design, area and period

**Fig. 1:**
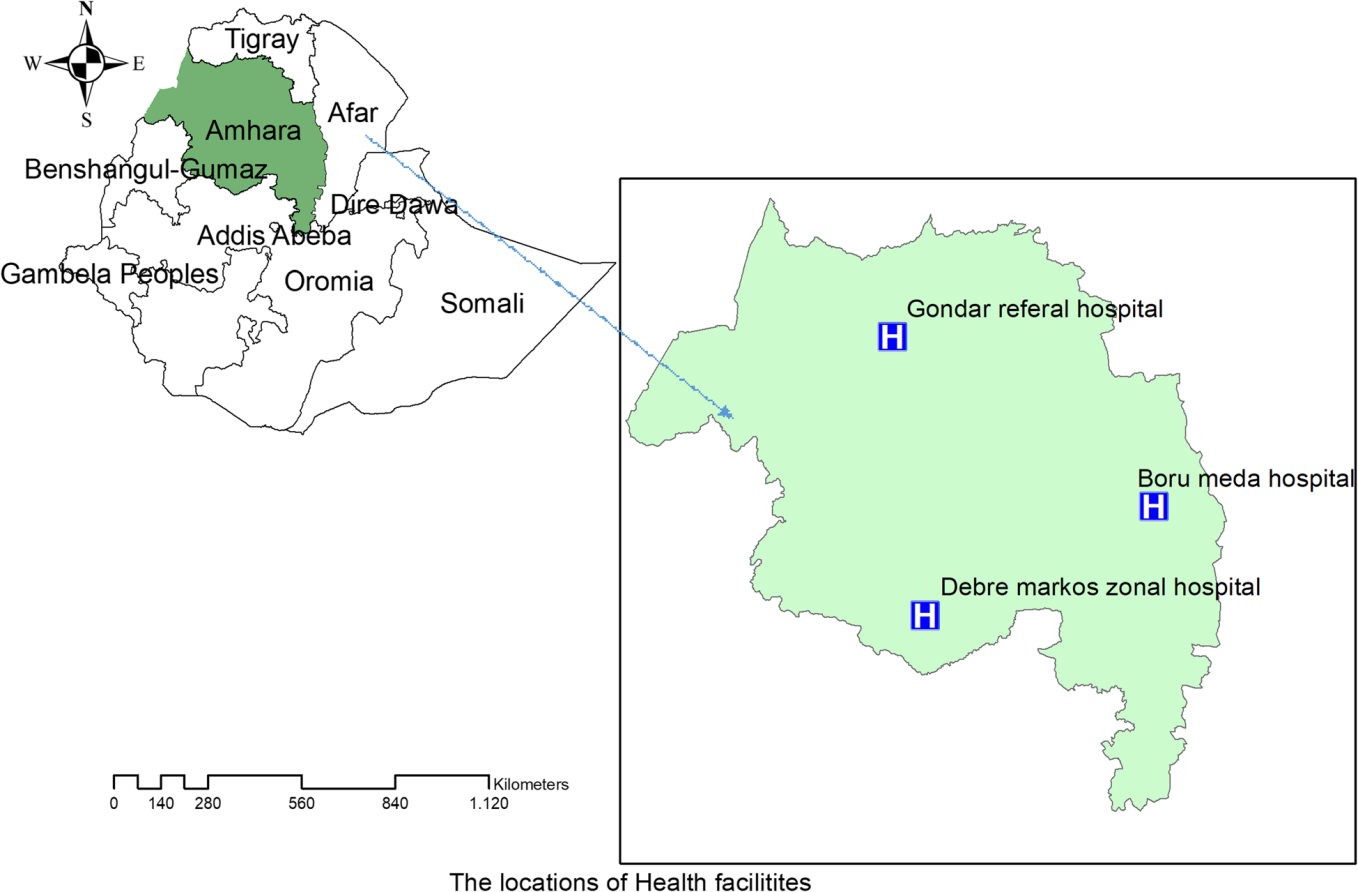
Map of the study settings (hospitals) in the Amhara Regional State, Ethiopia

An institution-based retrospective study was conducted at the University of Gondar comprehensive specialized and Borumeda and Debremarkos referral hospitals from September 2016 to December 2016. In the Amhara region, there are nine drug-resistant tuberculosis treatment initiating centers. The hospitals were selected because they are situated in the region’s main cities serving more than 85% of them. The remaining six centers included recently were located in the districts with the main purpose of supporting the three main TICs (hospitals) mentioned above as referrals for outpatient follow-ups and enhancing the accessibility of health services (Fig. 1). Stable clinical conditions, satisfactory adherence to DR-TB medications, and sputum culture conversions were the criteria used to discharge patients from the hospitals for outpatient follow-ups.

GeneXpert MTB/RIF Assay (GeneXpert® System – Cepheid), Line probe Assay (LPA), liquid (MGIT), and solid culture medium (LJ standard medium) were laboratory methods used to confirm a drug-resistant pattern of first-line anti-TB drugs. There was no LPA test for the second-line anti-TB medications during the study period, and the mentioned LPA result in this document was for first-line drugs. The sputum sample referral and laboratory result communication made through the postal office between reference laboratories and district health facilities to improve the accessibility of services [29]. The referral system between the foremost hospitals and district health facilities is a bidirectional patient diagnosed with the disease referred to these hospitals for treatment initiation and critical evaluation. When the patient met discharge criteria treatment and follow-up arranged at the district health facility.

### Population and sample

Patients admitted and discharged from the selected hospitals during the initial phase of MDR-TB treatment were the study population. The single population proportion formula with the assumptions of 50% of patients experienced more extended hospital stay, a 5% level of precision, and a 10% contingency rate for those with no treatment outcomes/discharge information. The final sample size became 422. A total of 490 multidrug-resistant tuberculosis patients were enrolled for DR-TB treatment in the three hospitals from September 2010 to December 2016, and we included 432 patients who fulfilled the inclusion criteria. Conversely, Fifty-eight patients were excluded due to incomplete data, death during treatment, and transfer before completing the intensive phase.

### Data collection procedures

The data available on patient records were first examined, and the appropriate extraction format was prepared in English. Six data collectors and two supervisors (nurses and health officers) were recruited. A two-day training was given on research objectives and document review using the data extraction format. Before the actual data collection process, records was identified by their medical registration numbers. The trained collectors reviewed and extracted data from patient charts and hospital admission/discharge logbooks using the checklists.

### Variables of the study

The dependent variable was the length of hospital stays in days from the date of admission to discharge. Whereas socio-demographic characteristics (sex, age, residence, housing condition, educational status, marital status), behavioral factors (smoking, alcohol use, khat chewing), and clinical characteristics (TB/HIV co-infection, registration group, a form of TB, presence of chronic diseases, adverse drug effects, radiological findings, treatment delay, baseline BMI, and functional status) were the independent variables. The length of hospital stays (LOS) refers to the duration of stays in days from the date of admission to the date of discharge under the initial phase of MDR-TB treatment with zero days of stay for a patient with less than 24 h of ward stay. When the patient stays admitted for more than a median cut off point, LOS ≥62 days are considered prolonged hospital stays,

Multidrug-resistant tuberculosis is defined as *Mycobacterium tuberculosis* strain resistant to the first-line drugs Isoniazid and Rifampicin, or when an individual is resistant only to Rifampicin and treated as multi-drug resistant [10]. A previously treated case was defined as a patient treated for TB for 1 month or more [23]. A patient with less than < 18.5 kg/m^2^ body mass index was classified as underweight, whereas a patient with ≥18.5 kg/m^2^ body mass index was classified as normal BMI. Treatment initiating centers (TIC) are health facilities selected by the TB program to provide patient care and treatment services from the time of DR-TTB diagnosis and throughout treatment with SLDs [9].

### Data analysis

Data were entered into EPI info version 7 and analyzed using Stata version 14 (StataCorp. 2015. Stata Statistical Software: Release 14. College Station, TX: StataCorp LP). Descriptive statistics such as frequencies, percentages, and median with interquartile range (IQR) was used to summarize categorical and continuous variables.

Based on LOS, patients were dichotomized using the median value, < 62 days (0) vs. 62 or more (1). The median of 62 days was used to categorize the patient’s length of hospital stay in two because there is no standardized cutoff point from previous studies. A binary logistic regression analysis used to identify factors associated with longer (62 or more days) hospital stays. Crude and adjusted odds ratio (OR) with 95% confidence intervals (CI) computed to assess the associations between socio-demographic and clinical factors and the prolonged hospital stays.

## Result

### Baseline socio-demographic characteristics

A total of 490 patients enrolled on standard MDR-TB treatment in the three hospitals. Fifty-eight patients excluded due to incomplete data, treatment initiated in an ambulatory model, death during treatment, and transfer before completing the intensive phase. A total of 432 patients were included in the final analysis.

Most of the admission/discharge 61.1%) were at the University of Gondar comprehensive specialized hospital, followed by Borumeda (28.2%) and the rest at Debremarkos referral hospital.

More than half (57.4%) of the patients were males, with the median age was 29 (IQR, 22 to 40 years), and 64.4% aged below 34 years. Of the participants, 43.1 and 34.3% were married and single, respectively; 56.9% had some primary and above educational level, while rural dwellers constituted 52.8% of the respondents. As far as substance use was concerned, 19, 12.7, and 8.3% drunk alcohol, smoked cigarettes, and chewed khat, respectively (Table 1).

**Table 1:** Socio-demographic and clinical characteristics of drug-resistant tuberculosis patients in Amhara region hospitals (*n* = 432)

Characteristics	Category	Frequency (%)
Age in years	≤24	142 (32.8)
	25–34	136 (31.5)
	35–44	82 (19)
	≥45	72 (16.7)
Level of education	No formal education	186 (43)
	Primary school	132 (30.6)
	Secondary school	75 (17.4)
	Diploma and above	39 (9)
Housing condition	Homeless	19 (4.4)
	Had housing	413 (96.4)
Occupation	Unemployed	171 (39.6)
	Government employed	39 (9)
	Private	187 (43.5)
	Student	35 (8.1)
Has treatment supporter	Yes	367 (85)
	No	65 (15)
HIV confection	Yes	319 (73.8)
	No	113 (26.2)
Diagnostic methods	GeneXpert Assay	203 (47)
	LPA	157 (36.3)
	Culture and DST	64 (8)
	Clinically	16 (3.7)
Baseline culture result (*n* = 430)	Positive	352 (81.5)
	Negative	20 (4.6)
	Unknown	60 (13.9)
Registration group	New	57 (13.2)
	Previously treated	375 (86.8)
Functional status at admission	Working	47 (10.9)
	Ambulatory	304 (70.4)
	Bed ridden	81 (18.7)
Body mass index (BMI) baseline	Low	115 (26.6)
	Normal	313 (72.5)
	Overweight	4 (0.9)
Base line Hgb in g/dl	< 7 g/dl	11 (2.5)
	7–9.9	49 (11.3)
	10–12.9	143 (33.1)
	> = 13	148 (34.3)
	Unknown	81 (18.8)
Adverse drug effects (ADE)	Yes	307 (71.1)
	No	125 (28.9)
Drug side effects (*n* = 307)	Gastro-intestinal upset	240 (78.2)
	Electrolyte disturbance	105 (34.2)
	Nephrotoxicity	25 (8.1)
	Psychosis	40 (13)
	Arthralgia	74 (24.1)
	Neuropathy	19 (6.2)
	Ototoxicity	8 (2.6)
	Others	14 (4.5)
Radiological findings	Cavitation	181 (41.9)
	Infiltrations	115 (26.6)
	Consolidations	78 (18.1)
	Chronic changes	107 (24.8)
	Others*	57 (13.2)

### Clinical characteristics

The pulmonary form of MDR-TB cases accounted for 93.5%, while the remaining were extrapulmonary cases. One-fourth (26.2%) of the MDR-TB patients 93.8% of whom were on ART had TB/HIV co-infections. One or more medical co-morbidities reported, involved 9.5% of the participants, of whom 2.8% had diabetes mellitus. One or more radiological abnormalities were seen on 71.3% of the patients. The most common radiological findings included 42.2% cavitation, 26.6% infiltration, and 24.7% chronic changes, like fibrosis. Out of the total patients, 71.1% had at least one adverse drug effect with gastro-intestinal upset, (81.5%) and electrolyte disturbance (33.6%), the most common side effects. Fifteen patients underwent surgery for tuberculosis related problems (Table 1).

Line probe assay (LPA) (45.6%) and GeneXpert (46.7%) were the most commonly used diagnostic methods for confirmation of drug-resistant TB. Concerning the TB resistance pattern, 96.3% of the patients had confirmatory drug resistance test results, 96% resistant to Rifampicin, and 45.6% to Isoniazid. Moreover, 8.3% of the patients were resistant to all first-line anti-TB drugs. The median time to start anti-TB medication was 10 (IQR of 3 to 72) days, and 70% of patients initiated treatment within 30 days after diagnosis (Table 2).

**Table 2:** Treatment outcome, median time to initiate treatment and length of hospitals stay by hospitals

Characteristics		Hospitals		
		University of Gondar	Borumeda	Debremarkos
Median length of hospital stay (days)		61 (35 to 100)	72.5 (47 to 110)	39 (24 to 70)
Median time to start treatment (days)		14 (3 to 72)	9 (2 to 18)	6.5 (2 to 15)
Treatment outcome	Cured	151 (57.8)	63 (50.8)	13 (27.6)
	Completed	14 (5.4)	1 (0.8)	3 (6.4)
	Unknown outcome	49 (18.8)	25 (20.2)	1 (2.1)
	Treatment failure	2 (0.8)	0 (0)	0 (0)
	On treatment (continuation)	45 (17.2)	35 (28.2)	30 (63.8)
Number of patients enrolled		261	124	47
Number of beds in the hospitals		21	14	14

### Length of in-hospital stays (LOS)

**Fig. 2:**
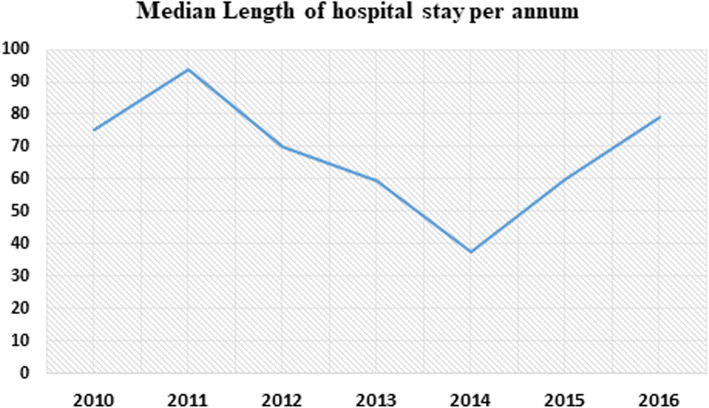
A line graph show median length of hospital stays over years among drug resistant tuberculosis patients in Amhara region hospitals from September 2010 to December 2016

The median duration of hospital stay during the intensive phase of MDR-TB treatment was 62 (IQR, 36 to 100 days), and the mean (SD) was 76.7 (±57.8) days. The median length of hospital stays for each treatment center, University of Gondar hospital was 59.5 (IQR, 34 to 100 days), Borumeda 72 (IQR, 47 to 111 days), and Debremarkos referral hospital 39.5 (IQR, 24 to76 days). Two hundred seventeen (50.7%) patients with a 95%CI (45.4. to 55.0) were hospitalized for longer than 62 days during the intensive phase of the MDR-TB treatment. The detailed description of LOS by socio-demographic and clinical characteristics presented on Table 3. Also, the median duration of hospitals stays per annum shown using the line graph in Fig. 2.

**Table 3:** Median length of hospital stay per clinical and socio-demographic characteristics of patients (*n* = 432)

Characteristics	Category	Median (IQR) LOS in days
HIV co-infection	Yes	71(34 to 112)
	No	59 (36.5 to 96)
Registration group	New	53 (32 to 78)
	Previously treated	36 (37 to 102)
Functional status at admission	Working	32 (14 to 80)
	Ambulatory	61 (37 to 89)
	Bed ridden	95 (47 to 137)
Baseline BMI	Low	62 (37 to 102)
	Normal	60 (35 to 93)
	Overweight	49 (28.5 to 84.5)
Chronic medical illness	Yes	80 (37 to 112)
	No	61 (36 to 99)
Adverse drug effects (ADE)	Yes	66(41 to 112)
	No	42(31 to 78)
Form of TB	Pulmonary	63.5 (37 to 105)
	Extra pulmonary	35.5 (14.5 to 53.5)
Age in years	< 24	56 (36 to 98)
	25–34	64 (33.5 to 97)
	35–44	63.5 (42 to 102)
	≥45	62 (32 to 122)
Housing condition	Homeless	112 (55 to 98)
	Had housing	61 (36 to 98)
Alcohol drinking	Yes	66.5 (34 to 120)
	No	61 (36 to 97)
Adverse drug effects	Gastro-intestinal upset	67.5 (41 to 112.5)
	Electrolyte disturbance	74 (41 to 157)
	Nephrotoxicity	70 (36 to 99)
	Psychosis	60.5 (38.5 to 97)
	Arthralgia	61 (37 to 111)
	Neuropathy	78 (51 to 126)
	Ototoxicity	77.5 (40.5 to 101.5)

### Factors associated with a prolonged hospital stay

The binary logistic regression model was fitted to identify factors associated with prolonged hospital stays (≥62 days). The pulmonary form of TB, bedridden functional status, and adverse drug effects were significantly associated with extended hospital stays at a *p*-value of 0.05 in the multivariable analysis model. Thus, patients who had a pulmonary form of drug-resistant tuberculosis, the odds of prolonged hospital stay, were 3.47 times higher than those of extrapulmonary cases (AOR = 3.47, 95%CI: 1.35 to 3.30). Similarly, patients who had a functional status of bedridden at admission had 2.88 times higher odds of prolonged hospital stays than those who had working functional status (AOR = 2.88, 95% CI: 1.29 6.43). Likewise, patients who had reports of adverse drug effects were 2.11 times more likely to have more extended hospital stays than those who didn’t have such adverse drug events (AOR = 2.11, 95%CI: 1.35 3.30) (Table 4).

**Table 4:** Binary logistic regression analysis to identify factors associated with prolonged hospital stays among drug resistant tuberculosis patients in Amhara region (*n* = 432)

Characteristics	Prolonged hospital stays		Crude OR (95%CI)	Adjusted OR (95%CI)
	Yes	No		
Age				
≤ 24	67	75	1	1
25–34	70	66	1.18 (0.74 1.90)	0.96 (0.58 1.59)
35–44	44	38	1.29 (0.75 2.23)	1.03 (0.57 1.88)
≥ 45	36	36	1.11 (0.63 1..97)	0.95 (0.52 1.74)
**Alcohol drinking**				
Yes	43	39	1.11 (0.68 1.80)	1.02(.61 1.70)
No	174	176	1	1
**Housing condition**				
Homeless	13	6	2.21 (0.82 5.95)	2.58 (0.87 7.66)
Had housing	204	209	1	1
**Presence of chronic illness**				
Yes	25	16	1.61 (0.83 3.12)	1.23 (0.60 2.49)
No	192	199	1	1
**Forms of TB**				
Pulmonary	211	136	4.0 (1.59 10.09)	3.47 (1.31 9.16)*
Extra pulmonary	6	22	1	1
**Adverse drug effects**				
No	46	79	1	1
Yes	176	136	2.15 (1.40 3.31)	2.11 (1.35 3.30)*
**Registration group**				
New	24	33	1	1
Previously treated	193	182	1.45 (0.83 2.56)	1.48 (0.80 2.76)
**Functional status**				
Working	16	31	1	1
Ambulatory	149	155	1.86 (0.97 3.54)	1.70 (0.86 3.36)
Bedridden	52	29	3.47 (1.63 7.39)	2.88 (1.29 6.43)*
**TB/HIV co-infection**				
Yes	65	48	1.48 (0.96 2.29)	1.23 (0.76 1.99)
No	152	167	1	1

* Shows statistical significance at a *p*-value of 0.05

## Discussion

In this study, the median length of hospital stay was 62 (IQR, 36 to 100) days. Bedridden functional status, the pulmonary form of TB, and adverse drug effects were factors associated with prolonged hospital stays (≥ 62 days). The length hospital stays also differed significantly between the hospitals, ranging from 39.5 days at Debremarkos referral hospital to 72.5 days at Bourmeda. The possible reasons might be the lack of consistent discharge criteria and professional expertise differences among hospitals. This shows high resource consumption, which makes hospitals less efficient, especially for facilities with limited beds and spaces.

The median length of hospital stay also explored over time, and differences observed from a median of 94 days in 2012 to 60 days in 2015. The possible explanations might be better patient management experience in the later time, the use of new treatment approaches, and early case detection through active surveillance before critical conditions happened might contribute to cutting LOS [30].

The median length of hospital stay in this work was shorter than the WHO 2014 global TB report of 90 days [22], South Africa centralized hospital of 144 days [23], South Africa community-based sites of 143 days [23], and Canada Ontario of 82 days [31]. Health care system differences, the clinical condition of the patients like the severity of the disease, and the presence of co-morbidities, like HIV and DM, might be responsible for the observed discrepancies. However, this attempt’s median length of hospital stay was longer than that of a study conducted in San Francisco, finding 14 days [13]. The possible reasons might be differences in treatment approaches; in the San Francisco study, MDR-TB treatment was provided through outpatient follow-ups, which decreased the length of hospital stays during treatments.

Thus, patients with the pulmonary form of tuberculosis associated with more extended hospital stay than extrapulmonary cases. The pulmonary form of the disease is clinically more symptomatic and associated with severe disease conditions, which might be related to more extended hospital stays. Also, pulmonary TB is more public health concern to the transmission of the bacilli to others. Thus, pulmonary TB patients came from congregated settings like university dormitories and prisons usually stay isolated in the hospitals until the patient had two consecutive negative sputum culture results. Moreover, the admissions of tuberculosis patients during the period of infectiousness are crucial for isolation, especially when patients came from congregated settings, like university dormitories [32], prisons and refugee camps where the risk of transmission is high. Before the revision of DR-TB treatment protocol, smear and culture-positive pulmonary drug-resistant tuberculosis patients stayed admitted until their sputum result was converted to negative, which could contribute to extended hospital stays among pulmonary TB sufferers [14, 28].

Similarly, bedridden patients at admission had three times higher odds of more extended hospital stays than patients who had working functional status. These might be due to the reasons patients with debilitating clinical conditions and altered functional states might have delayed clinical and treatment responses. Also, patients with low functional status have co-morbidities, like HIV co-infection, which causes advanced diseases that require more prolonged physician monitoring [15]. As shown in Table 3, the median length of hospital stay for working and bedridden functional status was 32 and 95 days, respectively, ultimately increasing resource utilization and hospital bed occupancies. Bedridden patients are highly dependent and are unable to self-care that could affect treatment follow-up.

Likewise, patients who reported having one or more adverse drug effects were two times more likely to stay longer at hospitals than those free form adverse drug effects. This finding is in line with studies conducted in Iran and Uzbekistan [33, 34]. Second-line anti-TB drugs are often more toxic and less effective. Some of the adverse drug effects are life-threatening and occult to detect; hence, more frequent and close follow up is mandatory for early detection and prompt treatment. As shown in the table, three adverse drug effects like neuropathy, ototoxicity, and electrolyte disturbance, the median LOS was 78, 77.5, and 74 days respectively. These toxicities are life-threatening and require close monitoring and follow up until improvements. Early anticipation and detection of adverse drug effects could reduce unnecessary prolonged hospital stays and saves the cost of treatment [35].

This study has implications for patients, healthcare workers, public health experts, health system administrators, and national tuberculosis programs to design efficient drug-resistant tuberculosis treatment protocol. Also, factors identified like bedridden functional status and adverse drug effects suggest the importance of early case detection and anticipated adverse events of SLD to reduce prolonged hospital stays that make health facilities more efficient. Furthermore, the findings of this study also helpful for evidence-based planning and resource allocation.

### Limitation of the study

Since this retrospective review was collected from secondary sources, some essential predictors, like adherence and health facility characteristics, which had significant associations with the length of hospital stay in other studies, were missing in the treatment of patients at the centers. Besides, baseline sputum culture and treatment outcome of some of the patents had unknown status due to poor documentation and patient file keeping. There were consistent no discharge criteria for in-patient management of DRTB and standard cut off point for the length of hospital stay and might introduce participants’ misclassification.

## Conclusion

The length of hospital-stay also differed significantly between the hospitals. Decreased functional status at admission, the pulmonary form of tuberculosis, and reported adverse drug effects were determinants of more extended hospital stays. These underscore the importance of early case detection and prompt treatment of adverse drug effects**.**

## Data Availability

Data is available from the corresponding author upon request because the data contains sensitive issues in the data set.

## Abbreviations

ADR: Adverse Drug Reaction
ART: Anti-Retroviral Therapy
BMI: Body Mass Index
DRTB: Drug Resistance Tuberculosis
DST: Drug Susceptibility Test
FLD: First Line Drug
GHC: Global Health Committee
HIV: Human Immune deficiency Virus
IQR: Inter Quartile Range
IRR: Incidence Rate Ratio
LOS: Length Of hospital Stay
LPA: Line Probe Assay
MDRTB: Multi-Drug Resistance Tuberculosis
MTB/RIF: Mycobacterium Tubercle Bacilli/Rifampicin
SLD: Second Line Drug
TB: Tuberculosis
TIC: Treatment Initiating Center
WHO: World Health Organization
XDRTB: Extensive Drug Resistance Tuberculosis
